# Anatomical Terminology

**Published:** 1908-09

**Authors:** 


### BOOK ON ANATOMY.

**Anatomical Terminology.** By Lewellys F. Barker, M.D. Pp. ix., 103. London: J. & A. Churchill. 1907.—The desirability of a uniform nomenclature in scientific matters is so obvious, that it should need no arguments to emphasise it. In the department of anatomy this ought now to be secured by the adoption of the terminology accepted at Basle by the German Anatomical Society. Its advantages are overwhelming. The terms are in Latin, thus rendering them intelligible all the world over, and synonyms are eliminated. A concession to terms associated with individuals is made by adding, in certain instances, the name in brackets. The Latin portion of the work under notice is the outcome of a Commission appointed by the Society in 1889, which consisted of Professors von Kölliker, O. Hertwig, His, Kollmann, Merkel, Schwalbe, Toldt, Waldeyer, and v. Bardeleben, with Professor W. Krause as editor-in-chief. The English equivalents have been prepared by Dr. Barker, with the help of Dr. Benson A. Cohoe, both members of the staff of Johns Hopkins Hospital. Ultra-conservatives will probably be able to offer many objections to the proposals. These seem to have been anticipated by the Commission, whose conclusions, and the reasons for them, are admirably set forth in an extremely lucid introduction which Dr. Barker has written for this volume. This introduction is full of interest, and well worth consideration even by those who are not anatomical specialists. Dr. Barker states that the new edition of Morris's *Anatomy* adopts the nomenclature. In course of time it ought to meet with universal acceptance.

